# Outcome of caesarean section at the Edward Francis Small Teaching Hospital, Banjul The Gambia

**DOI:** 10.4314/ahs.v18i1.20

**Published:** 2018-03

**Authors:** Patrick Idoko, Matthew Anyanwu

**Affiliations:** 1 Edward Francis Small Teaching Hospital, Banjul The Gambia; 2 School of Medical and Allied Health Sciences, University of The Gambia

**Keywords:** Caesarean section, Banjul The Gambia

## Abstract

**Background:**

Caesarean section is a very important procedure to decrease maternal and perinatal morbidity and mortality. Anecdotal evidence suggests that more than half of all caesarean sections done in The Gambia are done at the Edward Francis Small Teaching Hospital.

**Objective:**

The aim of the study was to determine the caesarean section rate at the Edward Francis Small teaching Hospital. The study also aimed to determine the socio-demographic factors associated with caesarean section and maternal and fetal outcomes of caesarean section at the hospital.

**Method:**

A retrospective review of all caesarean sections carried out at the Edward Francis Small Teaching Hospital from 1^st^ January 2014 to 31^st^ December 2014 was done. Data was extracted from patients' record. Descriptive statistics was done using Epi Info 7 statistical software.

**Results:**

The Caesarean section rate in the hospital is 24.0%. The commonest indications for caesarean section were previous caesarean section (20.6%) and cephalopelvic disproportion (20.2%). There were 21 maternal deaths (1.8%) and 71 fresh stillbirths (6.0%) in the study population.

**Conclusion:**

About a quarter of all deliveries in the hospital were caesarean sections most of which were done as emergencies. The commonest indications for caesarean section were cephalopelvic disproportion and previous caesarean section.

## Introduction

Caesarean section, a surgical procedure to deliver a baby in which an incision is made on the maternal abdomen and a second one on her uterus is an important tool for reducing maternal and perinatal morbidity and mortality. The exact origin of the procedure is shrouded in mystery and controversy. Before the later half of the 19^th^ century, the procedure was associated with almost certain maternal mortality^1^. However, with the advent of safe anaesthetic techniques, aseptic procedure and antibiotics, caesarean section has become a veritable tool in the armamentarium of physicians. In the past 30 years, the World Health Organisation (WHO) has evolved from recommending an ideal caesarean section rate of 10–15% to recommending that caesarean section be provided to all women in need rather than striving to achieve a specific rate^2^,^3^. Caesarean section rates higher than 10% are not associated with reductions in maternal and newborn mortality rate^3^.This change was due to emerging evidence of the benefits and risks of caesarean section along with significant improvements in clinical obstetric care^4^.

Over the years, as the procedure has become safer, the indications for the surgery have also changed. Historically, maternal health was the most important consideration in performing a caesarean section. More recently, the indication for caesarean section has expanded to include foetal conditions and maternal request^1^,^5^,^6^. About half of the caesarean sections performed in the United States are thought to be medically unnecessary^7^.

The medical consequences of a rising caesarean section rate remain uncertain in the short and long term and the implications in developing countries may be more significant as the facilities and/or capacity to properly conduct safe surgery and treat surgical complications may be limited^8^,^9^. A previous WHO study found that the rate of caesarean delivery was positively associated with adverse outcomes such as postpartum infection, postpartum antibiotic treatment and severe maternal morbidity and mortality, even after adjustment for risk factors^10^.

Even though caesarean section is much safer than it used to be in the past, this should not be taken to mean that the procedure is not fraught with many complications. The risk of maternal death is higher for caesarean section compared to vaginal deliveries^7^,^11^. It is also associated with higher morbidities including infection, haemorrhage and damage to surrounding organs^12^–^14^. Other problems include prolonged hospital stay, delayed mother-child bonding and higher hospital cost^7^. Respiratory distress syndrome is also more common in new born delivered by elective caesarean section before 38 completed weeks of gestation^11^. Subsequent pregnancies following a caesarean section are also associated with increased risks including unexplained stillbirths^15^,^16^.

As the only tertiary hospital in the country, the Edward Francis Small Teaching Hospital (EFSTH) receives referral from all over The Gambia. It also serves as the apex teaching hospital in the country and is involved in the training of doctors. Maternity care in The Gambia is absolutely free and provided for by the government. The aim of this study was to determine the caesarean section rate at the hospital as well as to determine the socio-demographic factors associated with caesarean section. The study will also determine the outcome of caesarean section at the EFSTH. Anecdotal evidence suggests that about half of all caesarean sections performed in the country are carried out at EFSTH. This study aims to determine the caesarean section rate at EFSTH, identify the socio-demographic factors associated with caesarean section and determine the maternal and foetal outcome of caesarean sections at EFSTH.

## Methodology

A descriptive cross sectional survey of all caesarean sections performed at EFSTH from 1^st^ January to 31^st^ December 2014 was done. The labour ward register and operating room register were used to identify all caesarean sections done during the study period. Data was extracted from patient's record on socio-demographic characteristics, indication for the surgery, maternal and perinatal outcomes and complications. Descriptive statistics with Epi Info 7 statistical software was used to analyze the data. Chi-square at significant level of 0.05 and confidence level of 95% was used to determine significance. Ethical approval was obtained from the ethics board of the Edward Francis Small Teaching Hospital, Banjul.

## Results

Of the 4900 deliveries, 1177 were caesarean sections giving a caesarean section rate of 24.0%. Table 1 shows the descriptive characteristics of parturients who had caesarean section compared with parturient who did not have caesarean section. The caesarean section rate for women with one previous caesarean section was 46.5%. Women with one previous caesarean section had a 2.75 - fold increased risk of a repeat caesarean section (p<0.0001).

**Table 1:** Socio demographic characteristics of study population

	Caesarean Section n = 1177 (%)	Vaginal delivery n = 3723 (%)	RR (95% confidence interval)
Mean age years (sd)	29.02 (7.02)	28.4 (6.7)	− 0.62* (−1.06 − −0.18) p = 0.0063

Marital Status			

Married Single Missing	1007 (85.6) 17 (1.4) 153 (13.0)	3488 (93.7) 23 (0.6) 212 (5.7)	0.91 (0.89 – 0.93) p = 0.0025

Educational level			

Primary or none Secondary and above Missing	776 (65.9) 184 (15.6) 217 (18.5)	1877 (50.4) 737 (19.8) 1109 (29.8)	1.30 (1.24 – 1.38) p < 0.0001

Parity			

0 1 – 4 > 4	436 (37.0) 590 (50.1) 151 (12.9)	894 (24.0) 1888 (50.7) 941 (25.3)	0.16 (0.12 – 0.21) p < 0.0001

Booking status			

Booked Unbooked Misssing	1017 (86.4) 18 (1.5) 142 (12.1)	3488 (93.7) 24 (0.6) 211 (5.7)	0.53 (0.37 – 0.75) p = 0.0018

Referral status			

Referred Not referred Missing	823 (69.9) 142 (12.1) 212 (18)	3350 (89.9) 194 (5.2) 179 (4.9)	0.78 (0.75 – 0.80) p < 0.0001

One previous Caesarean section			

Yes No	194 (16.5) 983 (83.5)	223 (6.0) 3500 (94.0)	2.75 (2.30 – 3.30) p < 0.0001

Mean Blood Loss in ml (sd)	410.5 (245.3)	217.9 (204.6)	−192.6* (−206.7 – −178.50) p< 0.0001

* difference in mean change

In table 2, the commonest indications for caesarean section were cephalo-pelvic disproportion and previous caesarean section.

**Table 2:** Indication for caesarean section

Indication for caesarean section	Frequency	Percentage
Previous Caesarean Section	242	20.6
Breech Presentation	184	15.6
Cephalo-pelvic disproportion	238	20.2
Failed Induction of labour	6	0.5
Eclampsia	47	4.0
Fetal Distress	59	5.0
Placenta Praevia	69	5.9
Obstructed labour	35	3.0
Slow progress in labour	67	5.7
Abruptio Placenta	69	5.9
Twin pregnancy	40	3.4
Cord Prolapse	20	1.7
Retained second Twin	20	1.7
Failed operative delivery	12	1.0
Ruptured uterus	19	1.6
Fetal macrosomia	12	1.0
Pre-eclampsia	19	1.6
Others	19	1.6

Table 3 shows the common complications following surgery. The minimum blood loss documented was 100 ml and the maximum was 985 ml with an average of 410 ml. The mean blood loss was higher in women with caesarean section compared to women who had vaginal delivery (Table 1) although the maximum blood loss recorded for spontaneous vaginal delivery was much higher at 2500 ml. However, 45.2% of the patients had blood transfusion about half of whom received more than 1 litre of blood. There were 21 maternal deaths (1.8%) in the women who had caesarean section (table 3).

**Table 3:** Complications following surgery

Complications	Frequency	Percent
Blood transfusion	532	45.2
Wound infection	51	4.3
Maternal death	21	1.8
Emergency Hysterectomy	27	2.3

* Hysterectomy was done for PPH in 25 cases and for severe endometritis in 2 cases

**Figure 1:**
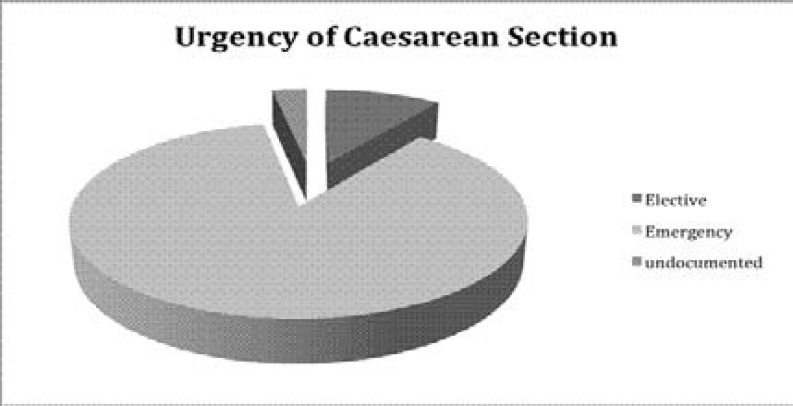
Urgency of Caesarean Section

**Figure 2:**
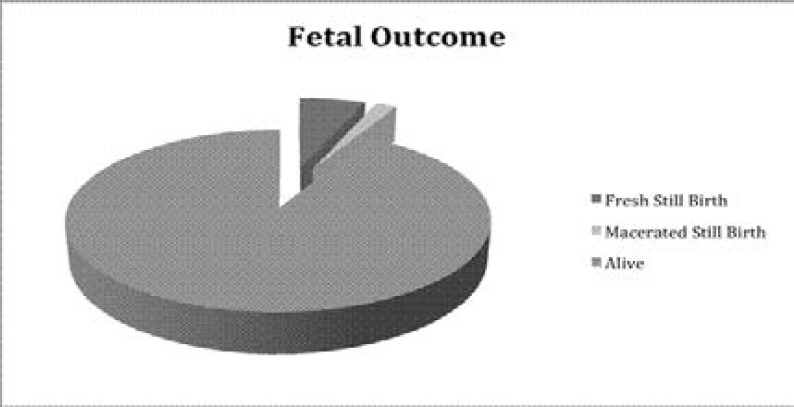
Foetal outcome following Caesarean Section

In figure 1, 87% of all caesarean sections done at the EFSTH were done as emergencies and figure 2 shows that while 6% of the caesarean section resulted in fresh stillbirth babies, macerated stillbirths were noticed in 2% of caesarean deliveries.

Table 4 shows the duration of hospitalization after surgery. The duration of stay after Caesarean section ranged from 1 day to 32 days with a mean of 4.5 days (standard deviation 3.6).

**Table 4:** Duration of hospitalization after caesarean section

Number of days	Frequency	Percent
1–3	617	53.4
4–7	437	37.1
8–14	78	6.6
≥15	33	2.9

## Discussion

The rate of caesarean section in this study was 24% that is a 1.5-fold increase from 14% in 2006 in the same hospital^17^. This invariably was beyond the estimated 10–15% rate suggested by the WHO as a recommended ideal rate^2^. While most Scandinavian countries have maintained this rate over the years^18^, there has been an overall increase in rates of caesarean delivery in many parts of the world^19^. In the United States, China and parts of South America caesarean section rates of 33 – 50% are common^20^–^22^. Similarly, evidence suggests that cesarean birth rates are high and increasing in some developing countries. A study conducted at a tertiary hospital in Nigeria suggests a caesarean section rate of 27.6% which was almost 3-fold more than the 10.4% recorded in this centre over two decades ago^23^. In a large population based survey conducted in 26 countries in Southern Asia or sub-Saharan Africa. The result shows wide regional variation of cesarean birth rates ranging from 3–26%^24^.

Studies have shown that the increased rate of caesarean delivery was driven by fear of litigation, caesarean section on maternal request and previous caesarean section^25^.

The mean age of women who had caesarean section was slightly higher than those who did not have caesarean section but this was not statistically significant (p = 0.0063). Previous studies have shown that advancing maternal age increases the risk for caesarean section^26^–^28^. This was not seen in this study, as most of the parturient were highrisk patients referred from other health centres. Being married reduced the risk of having a caesarean section in our study (RR 0.91, p = 0.0025). This is most likely due to the fact that married women are more likely to book for antenatal care where risk assessment would have been done and managed appropriately which may reduce the need for a caesarean section.

People with no formal education or just primary school level education were 1.30 times more likely to have a caesarean section than their more educated counterparts as shown in Table 1(p < 0.0001). While some previous studies have shown that women with higher education or higher socioeconomic status were more likely to have a caesarean section^29^–^31^, other studies have shown the opposite^32^,^33^. The EFSTH is a public health facility that offers free comprehensive maternity care. Thus, majority of the patients are from the lower socio-economic group with minimal formal education.

Caesarean section rates increased with increasing parity in our study (p <0.001). A Nigerian study found increased caesarean section rates for grand multiparous women^34^. Women who booked for antenatal care were less likely to have a caesarean section in this study (p = 0.0018). This is most likely due to the fact that obstetric risk assessment would have been done in women who booked and adverse pregnancy risks appropriately managed to reduce maternal morbidity. However, a study from Lagos, Nigeria did not find any association between booking for antenatal care and caesarean section^35^.

In our study, previous caesarean section (20.6%) and cephalopelvic disproportion (CPD) (20.2%) were the commonest indications for caesarean section. Women who had had a previous caesarean section were 2.75 times more likely to have a repeat caesarean section in this study (p < 0.0001). This is in keeping with previous studies^26^,^36^. Most of the caesarean deliveries done in the study were emergency procedures (87%). This is in keeping with several West African Studies that have shown that emergency caesarean deliveries were performed more commonly than elective procedures^13^,^35^,^37^. The outcome for elective caesarean section is usually better than emergency surgery. Therefore, more effort is needed to decrease the rate of emergency procedures through antenatal obstetric risk assessment.

Regarding complications of caesarean section post surgery, blood transfusion was the commonest post-operative complication (45.2%) followed by wound infection (4.3%) and emergency hysterectomy (2.3%). In a similar tertiary hospital based study conducted elsewhere, anaemia was the commonest post-operative complication, occurring in 32.5% women, followed by pyrexia 24% and wound infection rate of 9%, blood transfusion rate was not reported^23^. In our study, we reported blood transfusion as proxy for anaemia, which was the commonest postoperative complication. Although the rate of blood transfusion did not correlate with the amount of blood loss at surgery (mean volume loss of 410ml) that may not preclude anaemia and postpartum haemorrhage irrespective of the amount of blood loss^38^. It is also recognized that surgeons often under estimate blood loss during surgery^39^–^41^. Therefore, given the need for blood transfusion in more than 45% of patients who had caesarean section in this study, we suggest that the blood loss may have been under estimated in most cases. However, unnecessary blood transfusion is also a possibility. The absence of pre-operative and post-operative haemoglobin concentration made it difficult to conclude either way and it is a limitation of the study.

Wound infection following caesarean section is a well-established complication in many studies and in different settings over the years. However, in our study we observed a 4.3% rate of wound infection which is less than 6–10% estimated in most reviews^42^. All caesarean sections at the study site had prophylactic antibiotics either before the abdominal incision or post-surgery for 5 days. However, the infection rate as estimated in the data collected was 4.3%, which may not preclude under-reporting as anecdotal evidence suggests a higher rate of post caesarean, wound infection. Nevertheless, post-caesarean section wound infection is a significant morbidity and deserves a call for the routine use of peri-operative antibiotics in patients and this has been found to be useful in other centres^43^.

A limitation of the study is the absence of anthropometric measures like height and weight that have been found to be predictors of caesarean section in other studies^35^,^36^. There was also a significant amount of missing information (5% – 30%) in the records.

## Conclusion

The rate of caesarean section in our setting of 24% is comparable to other studies in the sub-region. The commonest indications were previous caesarean section and cephalopelvic disproportion. About 90% of caesarean sections were done as an emergency. Low educational status, being single, increasing parity and failure to book for antenatal care all increased the risk of having a caesarean delivery in The Gambia.
